# Allogeneic Hematopoietic Stem Cell Transplantation for Mixed or Overlap Myelodysplastic/Myeloproliferative Disorders

**DOI:** 10.3389/fonc.2022.884723

**Published:** 2022-08-05

**Authors:** Argiris Symeonidis, Spiros Chondropoulos, Evgenia Verigou, Vasileios Lazaris, Alexandra Kourakli, Panagiotis Tsirigotis

**Affiliations:** ^1^ University of Patras Medical School, Hematology Division, Patras, Greece; ^2^ University General Hospital Attikon, Athens, Greece; ^3^ Hematology Division, General University Hospital of Patras, Rion of Patras, Greece; ^4^ Department of Medicine, School of Health Sciences, National and Kapodistrian University of Athens, Athens, Greece

**Keywords:** allogeneic stem cell transplantation, chronic myelomonocytic leukemia (CMML), atypical chronic myelogenous leukemia (aCML), Juvenile Myelomonocytic Leukemia (JMML), chronic neutrophilic leukemia (CNL), outcome, prognosis

## Abstract

Chronic myelomonocytic leukemia (CMML) and the remaining, less frequent hybrid, mixed, or overlap myelodysplastic syndromes/myeloproliferative neoplasms (MDSs/MPNs) are difficult to treat neoplastic hematological disorders, exhibiting substantial clinical and prognostic heterogeneity, for which clear therapeutic guidelines or effective treatment options are still missing. CMML has an overall survival ranging from a few months to several years. Although patients with proliferative or dysplastic features may benefit from hydroxyurea and hypomethylating agent treatment, respectively, none of these treatments can establish long-term remission and prevent the inevitable transformation to acute leukemia. Novel targeted treatment approaches are emerging but are still under investigation. Therefore, currently, allogeneic stem cell transplantation (allo-SCT) remains the only treatment modality with a curative potential, but its widespread application is limited, due to significant morbidity and mortality associated with the procedure, especially in the elderly and in patients with comorbidities. Recognition of patient eligibility for allo-SCT is crucial, and the procedure should be addressed to patients with a good performance status without severe comorbidities and mainly to those in intermediate- to high-risk category, with a suitable stem cell donor available. The issues of best timing for performing transplantation, patient and donor eligibility, the type of conditioning regimen, and the outcomes after various allo-SCT procedures are the topics of this review.

## Introduction

Myelodysplastic syndromes/myeloproliferative neoplasms (MDSs/MPNs) represent a difficult-to-treat group of clonal hematopoietic stem cell disorders, without specific molecular signatures, exhibiting both, myelodysplastic and myeloproliferative features. According to the most recent revision of the World Health Organization (WHO) classification, entities classified in this category include chronic myelomonocytic leukemia (CMML), atypical bcr/abl-negative chronic myeloid leukemia (aCML), juvenile myelomonocytic leukemia (JMML), and myelodysplastic syndrome/myeloproliferative neoplasm unclassifiable (MDS/MPN-U) (1, 2). Chronic neutrophilic leukemia (CNL), although currently classified among Myeloproliferative Neoplasms (MPNs), sometimes shares several dysplastic features, and it has been postulated that this disease might stand closer to MDS/MPN (3, 4).

For the most common entity, CMML, several prognostic systems have been proposed to best stratify patient life expectation, according to disease aggressiveness. French-American-British (FAB) Classification has defined the threshold of 13 × 10^9^/l white blood cells (WBCs) to distinguish the dysplastic from the proliferative subtype, but this cutoff value cannot reflect the biological differences of the two subtypes. Cytogenetic risk, appears not to be comparable to that of classical MDS (5). Prognostically relevant is the WHO 2016 classification, based on the percentage of peripheral blood (PB) and/or bone marrow (BM) blasts, as CMML-0 (BM blasts 0%–4%), CMML-1 (5%–9%), and CMML-2 (10%–19%), although often clear differences between CMML-0 and CMML-1 may not be found. However, difficulties might emerge in the correct characterization of marrow blasts, since several immature monocytoid cells could be considered blasts, as also promonocytes should be considered as blasts, together with myeloblasts and monoblasts. Thus, this concept should always be kept in mind when assessing patient risk according to the WHO subclassification (CMML-0 vs. CMML-1 vs. CMML-2) and/or according to scoring systems such as the CMML Prognostic Scoring System(CPSS) including BM blast percentage among prognostic factors to be considered (6). Other systems, such as the MD Anderson Prognostic Score (MDAPS) and the Mayo prognostic model, rely on clinical, morphological, and laboratory parameters (7, 8) because either they did not test the importance of genetic markers (7) or the tested markers were not proven to be prognostically important (8). Molecular information has been incorporated within the Group Francophone (GFM) (9), the Mayo, and the CMML Prognostic Scoring System molecular (CPSS-mol) (10) prognostic systems, in the latter together with cytogenetics as genetic risk grouping. In a comparative study between the CPSS, the MDAPS, and the Mayo prognostic system, CPSS was found superior, and the authors further improved it by adding platelet count, thus creating the CPSS-P (11). However, all of these tools are applicable at baseline, and not to patients proceeding to allogeneic stem cell transplantation (allo-SCT). BM fibrosis may occur in some CMML patients, who more frequently exhibit Janus Kinase 2 (JAK2) gene mutations. Patients with fibrotic CMML have a dismal outcome and should be distinguished from patients with primary myelofibrosis and monocytosis (12). Finally, therapy-related CMML appears to be pathogenetically distinct, has worse prognosis than primary disease, and has also been suggested to be distinguished (13, 14).

## Overview of Treatment Approaches for Chronic Myelomonocytic Leukemia and the Other Myelodysplastic Syndromes/Myeloproliferative Neoplasms

Treatment options for these diseases vary from supportive care to allo-SCT. This variability clearly reflects the extreme heterogeneity of prognosis, according to disease and patient characteristics at diagnosis. Until recently, clear treatment guidelines were lacking, although excellent reviews and treatment recommendations have been published (15–18). The lack of evidence-based recommendations is mainly attributed to the absence of large, multicenter, prospective, randomized trials investigating prespecified treatment outcomes. A potential explanation is the high degree of clinical, laboratory, molecular, and prognostic heterogeneity of these diseases. The use of hypomethylating agents (HMAs) as initial treatment becomes more and more popular; however, results are less favorable than those achieved by patients with classical MDS, fewer patients achieve complete remission (CR), and responses are shorter (19, 20). Recent molecular analyses have shown that ASXL1 and RAS mutations are associated with poorer response to HMA treatment, whereas TET2 mutations represent a favorable factor for response (21).

Oral cytoreductive treatment, usually hydroxyurea, is temporarily effective and is administered to patients with the proliferative subtype or with extramedullary organ involvement. Single-agent chemotherapy, most commonly low-dose subcutaneous or intravenous cytarabine, may be given to patients with uncontrolled monocytosis and/or increased marrow blasts. However, responses are short, and the majority of patients soon become refractory, developing multidrug resistance. Combination chemotherapy, consisting of cytarabine plus an anthracycline or a topoisomerase inhibitor, may be more effective, induces longer remissions, but is poorly tolerated by the usually advanced-aged or unfit patients. Combination chemotherapy-induced CR is usually of short duration, as compared to remissions induced in patients with *de novo* AML.

The only treatment with curative potential remains allo-SCT, which should be provided to all patients with prognostically unfavorable, rapidly evolving, or symptomatic CMML, or other MDSs/MPNs, who have an available stem cell donor. Ideally, this should be performed early, before disease progression, because in the latter case, non-relapse mortality (NRM) and relapse rate (RR) are higher and worse than those in AML evolved from classical MDS (22). There are several barriers, preventing the broad application of allo-SCT, including patient-related issues (advanced age, poor performance status, comorbidities) and disease-related issues (unstable disease, delayed postchemotherapy marrow reconstitution, infectious complications, and organ impairment).

Several studies aim to rationalize the therapeutic decision for allo-SCT in these diseases. According to the Mayo Clinic experience, among 406 patients, 70 underwent allo-SCT, and median leukemia-free survival (LFS) by the application of propensity score-matched analysis was clearly better in the transplanted than that in the non-transplanted group (40 vs. 20 months) as was overall survival (OS; 40 vs. 21 months) (23). Furthermore, in a multicenter analysis of 261 patients, of whom 119 underwent allo-SCT, after a prolonged median follow-up of 6.1 years, transplanted patients had significantly better median OS (4.3 vs. 2.3 years) (24).

## Experience From Allogeneic Stem Cell Transplantation in Patients With MYELODYSPLASTIC SYNDROMES / MYELOPROLIFERATIVE NEOPLASMS

### A. Chronic Myelomonocytic Leukemia

The existing experience is restricted to retrospective analyses from various transplant groups. However, in many retrospective studies, results of allo-SCT in patients with CMML are pooled together with the results of patients with other myeloid malignancies (other MDSs/MPNs, MDS, AML, MPN, etc.), and only exceptionally the outcomes of CMML patients are mentioned separately. Most recently, however, some retrospective studies have focused on the outcomes of CMML and occasionally on other MDSs/MPNs.

In early reports from the MD Anderson Cancer Center (MDACC) on 20 patients (8 with CMML), probabilities for disease-free survival (DFS) and OS at 2 years were 37% and 47%, respectively (25), while from the Mayo Clinic, among 17 transplanted patients <60 years, 7 were relapsed and, in 6 of them, 1–3 courses of donor leukocyte infusions (DLIs) were offered. Despite the high treatment-related mortality (TRM) of 41% at 3 years, 3 patients remained alive, indicating a graft-versus-leukemia (GVL) effect (26). In support of a GVL effect was the first analysis of the European Blood and Marrow Transplantation Registry (EBMT) on 50 patients, who, despite a 52% TRM at 5 years, demonstrated a lower probability of relapse when grade II–IV acute graft-versus-host disease (GVHD) developed (24% vs. 54%) and higher RR when patients received T cell-depleted allografts (27). Among 148 patients, who received a reduced-intensity conditioning (RIC), after a median of 47 months, relapse-free-survival (RFS), OS, and RR at 3 years were 27%, 27%, and 41%, respectively. For 7 CMML patients, RFS and OS were both 43% (28). Initial experience from Hamburg on 12 patients was also positive, with a low TRM, 50% DFS, and 75% OS at 4 years (29). At the King’s College, 18 CMML patients received an RIC and T cell-depleted allografts. The probabilities of OS, RR, and TRM at 3 years were 31%, 47%, and 31%, respectively (30). Among 21 CMML patients transplanted in the Fred Hutchinson before 2000, 9 achieved sustained Disease-Free Survival (DFS) after a median of 7 years (31), and in a later analysis of 43 patients, NRM, RR, RFS, and OS at 4 years were 35%, 23%, 41%, and 41%, respectively (32). In the Mayo Clinic, out of 43 patients (35 with CMML, 17 on AML status), after a median of 21 months, NRM, RR, and OS were 25%, 29%, and 55%, respectively, for patients transplanted in the chronic phase and 34%, 40%, and 47%, respectively, for those transplanted following AML transformation (33). Similar results were confirmed on 70 patients, of whom 46 were transplanted in the chronic phase and 24 after AML transformation. Median OS was better for patients transplanted in the chronic phase (67 vs. 16 months), and Kaplan-Meier (K-M) estimates for OS at 5 years was 51%—one of the best ever reported (23).

In the French retrospective study of 73 CMML patients, OS, NRM, Event-Free Survival (EFS), and RR at 3 years were 32%, 36%, 29%, and 35%, respectively. NRM was lower in female patients, those transplanted after 2004, and in patients without palpable splenomegaly or pretransplant infections (34). Another collaborative analysis of 85 CMML patients, including 14 with therapy-related disease, reported 25% RR at 3 years, and the use of myeloablative conditioning was associated with better outcomes, compared to RIC (35). In the analysis from MDACC on 83 patients, the 12-month TRM was 31%, and patients who were bridged with HMA had lower RR at 3 years, compared to those receiving AML-type induction chemotherapy (22% vs. 35%), resulting in significantly longer PFS (43% vs. 27%) (36). A German group report on 45 patients, mainly with CMML, observed a low 3-year NRM of 26.7%, while OS at 5 years was 51%. The presence of mutations was used as a marker of minimal residual disease, and their persistence 6 months posttransplant was associated with significantly higher RR (37). The Nordic group applied a *post-hoc* analysis on 51 CMML patients, with a median follow-up of 5.5 years, and identified clonal mutations in 48 of them. ASXL1, TET2, RUNX1, SRSF2, and RAS were the most frequently mutated genes. Transplantation outcomes were better than those previously reported, with a 5-year OS of 46.5%, NRM of 30%, and RR of 25% (38).

The impact of the donor was investigated on 159 Japanese patients. OS, NRM, and RR at 3 years were 33%, 28%, and 39%, respectively, and the best OS was obtained by [(MRDs), 50.4%], followed by matched-unrelated donors (MUDs, 31.4%), umbilical cord blood (UCB, 15.4%; TRM >75%), and mismatched-unrelated donors (MMUDs, 16.7%) (39). The Fred Hutchinson group described outcomes on 129 patients with the longest median follow-up (9.3 years). Estimated probabilities for relapse, DFS, and OS at 10 years were 32%, 29%, and 30%, respectively, whereas NRM was 32% (40).

Many studies have focused on the conditioning regimen, and majority of them do not report any impact, with the exception of one small study on 10 patients, in which myeloablative conditioning was associated with longer EFS (41). The same is reported by the Heidelberg group on 44 patients, in whom intermediate total body irradiation (TBI) dose (6–8 Gy) combined with mofetil mycophenolate posttransplant immunosuppression was associated with longer LFS in the elderly and less fit patients, compared to alkylator-based conditioning (42). A treosulfan-fludarabine regimen, although administered to older patients with comorbidities, was accompanied by better OS than standard myeloablative or RIC regimens (43). The addition of 2 Gy TBI over a standard treosulfan-fludarabine regimen was investigated on 51 patients with MDS and 49 with CMML. The TBI regimen showed superiority and was associated with longer PFS, whereas NRM was only 9% (44). In another prospective study on 77 patients (13 with CMML), a three-level dose-escalation TBI at non-myeloablative doses (300–450 cGy) was tested. RR, NRM, PFS, and OS at 5 years were 31%, 43%, 35%, and 38%, respectively (45). Total lymphoid irradiation (TLI) and anti-thymocyte globulin (ATG) were used in Stanford for 61 patients, and NRM at 3 years was only 11%, whereas PFS and OS were 35% and 41%, respectively. The authors recommend this regimen for patients with more advanced age (46).

The International Blood and Marrow Transplantation Registry (IBMTR) and the EBMT have published the largest retrospective studies. In the first, 209 patients were transplanted between 2001 and 2012, 35% of them receiving a graft from MRD and 27% exhibiting >5% marrow blasts. The type of bridging treatment (HMA vs. chemotherapy) and the type of conditioning (myeloablative vs. RIC) had no impact on outcome. TRM, RR, DFS, and OS at 5 years were 28%, 52%, 20%, and 30%, respectively (47). In the second, 513 patients who received a related (55.5%) or an unrelated graft (44.5%) following myeloablative conditioning (48.5%) or RIC (44%) were transplanted until December 31, 2009. Disease status at transplantation was CR in 24% and no CR in 67%. NRM, RR, DFS, and OS at 4 years were 41%, 32%, 27%, and 33%, respectively (48).

There is only one study describing encouraging outcomes with haploidentical transplantation on 19 CMML patients. The incidence of acute and chronic GVHD was acceptable, the 3-year TRM was 27%, and RR was only 11%, whereas LFS and OS were 57% and 64%, respectively. The authors suggest that this type of allo-SCT might exert a stronger GVL effect, and hence, RR may be low (49). A summary of the published reports on CMML, with the main patient and transplantation features and outcomes, is presented in **Table 1**.

**Table 1:** Published retrospective studies reporting transplantation outcomes on CMML.

Year	Group	1st Author/ Reference	All pts	CMML pts	Other pts	Med Age	♂/♀	Disease status at SCT (N,%)	RIC (%)	Related Donor n (%)	Med F-up (mo)	aGVHD N (%)	cGVHD N (%)	NRM (%)	RR (%)	DFS/RFS (%)	OS (%)
2000	Fred Hutch	Zhang (31)	21	21	0	47	14/7: 2.0	CP: 9 (43) EB: 12 (57)	0	12 (57)	83.0	15/21 (71)	14/16 (87)	33	25	3-yr 39	5-yr 43
2002	EBMT	Kröger (25)	50	50	0	44	29/21: 1.4	CP: 32 (64) BP: 18 (36)	N/A	38 (76)	40.0	28/48 (59)	7/27 (26)	52	49	5-yr 18	5-yr 21
2004	MDACC	Mittal (25)	20	8	7	51	15/5: 3.0	Not reported	0	15 (75)	17.5	9/20 (45)	12/20 (60)	30	31	2-yr 37	2-yr 47
2005	Fred Hutch	Kerbauy (32)	43	43	0	48	25/18: 1.4	CMML1: 32 CMML2: 11	2 (5)	18 (42)	69.0	N/A	21/39 (54)	35	23	4-yr 41	4-yr 41
2006	Mayo Clinic	Elliot (26)	17	17	0	50	11/9: 1.2	CP: 9 (53) BP: 8 (47)	1 (6)	14 (82)	34.5	12/16 (75)	6/15 (40)	41	41	3-yr 18	3-yr 18
2009	Hamburg	Ocheni (29)	12	12	0	56	5/7: 0.7	CR: 2 (17) no-CR: 10 (83)	6 (50)	2 (17)	26.0	10/12 (83)	5/10 (50)	25	17	2-yr 67	2-yr 75
2010	King's College	Krishnamurty (30)	18	18	0	54	12/6: 2.0	CR: 8 (44) no-CR: 10 (56)	15 (83)	7 (39)	16.0	8/18 (44)	3/14 (21)	22	47	3-yr 31	3-yr 31
2011	Fred Hutch	Eissa (35)	85	85	0	52	52/33: 1.6	CMML1: 57 CMML2: 26	15 (18)	34 (40)	62.0	58/81 (72)	37/84 (44)	33	27	10yr 38	10yr 40
2013	French	Park S (34)	73	73	0	53	NR	CR: 23 (31) no-CR: 50 (69)	43 (59)	41 (56)	23.0	28/68 (41)	25/71 (35)	36	35	3-yr 35	3-yr 36
2013	German	Fu (37)	45	39	6	57	25/20: 1.3	Not reported	30 (67)	10 (22)	46.0	28/45 (62)	22/40 (55)	27	41	5-yr 34	5-yr 46
2013	Korean	Lim (41)	10	7	3	43	9/1: 9.0	CR: 1 CP: 2, no-CR: 7(70)	5 (50)	5 (50)	47.5	2/10 (20)	4/9 (44)	10	40	5-yr 47	5-yr 42
2015	EBMT	Symeonidis (48)	513	513	0	53	343/170: 2	CR: 122(26) no-CR: 344(74)	226 (40)	276 (54)	43.0	155/470 (33)	123/374(33)	41	32	4-yr 27	4-yr 33
2016	MDACC	Kongtim (36)	83	83	0	57	58/25: 2.3	CR: 24 (29) no-CR: 59 (71)	19 (23)	30 (36)	48.0	27/75 (36)	27/72 (37)	31	33	3-yr 34	3-yr 35
2017	Mayo Clinic	Sharma (33)	43	35	8	55	24/12: 2.0	CP: 18 (51) EB: 17 (49)	21 (49)	19 (54)	21.0	26/35 (74)	22/32 (69)	25-34	29-40	NR	55 vs 47
2017	IBMTR	Duong Liu (47)	209	209	0	57	146/63: 2.3	CR: 136 (65) no-CR: 73 (35)	99 (48)	73 (35)	51.0	76/209 (36)	98/209 (47)	28	52	5-yr 20	5-yr 30
2018	Japanese	Itonaga (39)	159	159	0	54	119/40: 3	CR: 25 (16) no-CR: 134 (84)	67 (42)	51 (32)	NR	NR	NR	28	39	NR	3-yr 33
2020	Fred Hutch	Woo (40)	129	129	0	55	85/44: 1.9	CMML0-1: 52 2-AML: 75	21(19)	38 (29)	88.0	93/126 (74)	57/126(45)	31	32	3-yr 37	3-yr 38
2020	Heidelberg	Radujkovic (42)	44	44	0	61	27/17: 1.6	CR: 14 (32) no-CR: 30 (68)	7 (16)	10 (23)	39.0	6/36 (17)	8/34 (23)	16	44	3-yr 38	3-yr 56
2020	Mayo Clinic	Pophali (23)	70	70	0	58	46/24: 1.8	CP: 46 (66) BP 24 (34)	37 (54)	28 (42)	70.0	29/63 (46)	41/63 (65)	29	27	NR	5-yr 44
2020	Chinese	Sun (49)	19	19	0	41	10/9: 1.1	CR: 3 (16) no-CR: 16 (84)	Beijing pr	Haplo	39.5	7/17 (39)	2/12 (17)	27	11	3-yr 57	3-yr 64
2021	Nordic	Wedge (38)	64	64	0	62.5	49/15: 3.2	CR: 18 (28) no-CR: 46 (72)	23 (38)	20 (31)	65.0	34/60 (56)	-57	30	25	NR	5-yr 46
2021	German	Gagelmann (24)	119	119	0	58	83/36: 2.3	CMML0/1: 65 (55)	63 (53)	26 (22)	73.0	NR	NR	30	27	5-yr 43	5-yr 50
2021	German	Gagelmann (50)	240	240	0	59	172/68: 2.5	CMML0/1: 143 (59.6)	134 (56)	50 (21)	66.0	NR	NR	NR	NR	NR	NR

### B. Juvenile Myelomonocytic Leukemia

JMML is a rare pediatric leukemia affecting 1.2 children per million annually, with a median age at diagnosis of 2 years and a clear male predominance. It has an aggressive clinical course with a median OS of 10–12 months (51). Main features include splenomegaly, lung and gastrointestinal system monocytic infiltration, a leukoerythroblastic peripheral smear with absolute monocytosis, elevated fetal hemoglobin (HbF), and a hypercellular BM with increased blast percentage but <20% (52). Nearly all JMML cases (90%–95%) harbor either somatic mutations of the RAS pathway genes (PTPN11, KRAS, NRAS) or germline mutations of NF1 and CBL, which are involved in two congenital development disorders, namely, neurofibromatosis and Cbl protooncogene – E3 ubiquitin protein kinase (CBL) syndrome (53). Noonan syndrome caused by germline mutations of PTPN11, NRAS, KRAS, BRAF, SOS1, and RAF1 may exhibit a JMML-like disorder that is usually self-limited (54). Age >2 years at diagnosis, platelets <33 x 10^9^/L, and HbF >10% have been identified as main predictors of poor survival (55).

In essentially all cases of JMML, allo-SCT is strongly indicated and ideally should be performed immediately after diagnosis. Cytoreductive strategies usually involve azacytidine or AML-type chemotherapy, while occasionally, splenectomy is performed for symptom alleviation. Since the patient population is composed of children, TBI is not usually included in the conditioning, but busulfan-based regimens are used. The European Working Group on MDS (EWOG-MDS) provides a recommendation for a three-alkylator regimen consisting of busulfan, cyclophosphamide, and melphalan (56). MRD or MUD should be the first option, while one-locus MMUD or UCB transplantation is a reasonable alternative. Recently, a large study from China compared 27 patients transplanted with an MRD or MUD (Cohort-1), with 20 patients who underwent allo-SCT by using an haploidentical or an MMUD with 2 or 3 HLA disparities (Cohort-2). With a median follow-up of 26 months, OS, DFS, and NRM were 66%, 55%, and 11%, respectively, in the entire group, but the cumulative RR was significantly increased in Cohort-1 as compared with Cohort-2 (56% vs. 5%, *P* ≤ 0.001). Nevertheless, haploidentical allo-SCT might represent a solution for patients with a rapidly evolving disease for whom an MRD or an MUD is not available (57).

Age at diagnosis >2 years, NF1 or PTPN11 mutation, and high DNA methylation define a patient group with an RR of >50%, raising the issue of immunosuppression intensity and posttransplant prophylaxis (58). Thus, EWOG-MDS recommends keeping immunosuppression with cyclosporine-A at low levels (~80 g/L) and tapering early (from day +40 in the absence of grade II–IV GVHD). Donor chimerism should be tested at very short intervals (even weekly in high-risk patients), since the reappearance of small autologous cell populations mandates immediate withdrawal of immunosuppression (59). Prevention of relapse by preemptive administration of azacytidine or DLI is a frequently applied strategy. Novel approaches such as MAP kinase–ERK kinase (MEK) inhibitors (trametinib) or bcl2 inhibitors (venetoclax) in combination with azacytidine and anti-CD47 monoclonal antibodies are currently evaluated in the context of clinical trials (60). **Table 2** presents the results of allo-SCT in patients with JMML (56, 57, 61–63).

**Table 2:** Allogeneic stem cell transplantation in patients with JMML.

Author (ref)	No	Donor	Conditioning	Disease phase	PFS	OS
**Lin Y** (57)	**47**	**MRD: 11** **MUD: 22** **Haplo: 14**	**MAC: 47**	**Chronic: 38** **Blastic: 9**	**54% (5-year)**	**66% (5-year)**
**Tufecki** (61)	**28**	**MRD: 18** **MUD: 8** **UCB: 1** **Haplo: 1**	**MAC: 28**	**NR**	**Relapse rate 35%**	**56% (5-year)**
**Locatelli** (56)	**100**	**MRD: 48** **MUD: 52**	**MAC: 100**	**Chronic: 77** **Blastic: 10** **Missing: 13**	**54% (5-year)**	**66% (5-year)**
**Locatelli** (62)	**110**	**UCB: 100**	**MAC: 100**	**Chronic: 100**	**44% (5-year)**	**52% (5-year)**
**Yoshida** (63)	**129**	**MRD: 44** **MUD: 85**	**MAC: 116** **RIC: 13**	**NR**	**46% (5-year)**	**64% (5-year)**

MRD, matched related donor; MUD, matched unrelated donor; MAC, myeloablative conditioning; RIC, reduced-intensity conditioning; PFS, progression-free survival; OS, overall survival.

### C. Atypical Chronic Myelogenous Leukemia and Unclassified Myelodysplastic Syndrome/Myeloproliferative Neoplasm

aCML mainly affects elderly patients of male predominance and is characterized by inherent propensity for AML transformation. This is a difficult-to-treat disease with the available conventional treatment options with a median OS of about 2 years from initial diagnosis. In a group of 73 patients, age >65 years, anemia (<10 g/dl) and severe leukocytosis (>50 × 10^9^/l) at diagnosis were recognized as significant adverse prognostic factors and have been used to construct a simple prognostic system, greatly affecting survival (64). In two cohorts of 55 and 65 patients from Italy and the United States, prognosis was generally poor and median OS was 25 and 12 months, respectively. AML transformation occurred in >30% of patients between 12 and 18 months from initial diagnosis [65, 66]. In a retrospective analysis of 65 patients from MDACC, intensive chemotherapy was poorly tolerated and was associated with significantly decreased OS, as compared to HMA, hydroxyurea, or ruxolitinib treatment. Recently, in a new multivariate analysis on 65 patients, older age, thrombocytopenia, increased BM blasts, and abnormal serum lactate dehydrogenase (LDH) were parameters independently associated with decreased OS. Based on these parameters, a new scoring system was generated for a more accurate prediction of survival (67). The mutational landscape of aCML mainly involves SETBP1 and ETNK1 genes, while other commonly identified mutations include ASXL1, N/K-RAS, SRSF2, and TET2 and less frequently (<10%) CBL, CSFR3, and EZH2. JAK2, CARL, and MPL mutations are extremely uncommon (68). SETBP1 mutations have been associated with severe anemia and thrombocytopenia, increased LDH, and worse OS (69, 70). Regarding allo-SCT, many questions related to the timing of transplantation, bridging therapy, donor type, and intensity of preparative regimen still remain unanswered. In a retrospective study of 60 patients with MPN or MDS/MPN in blastic phase from the French Registry, with many of them receiving intensive cytoreduction as bridging treatment, 26 were in CR before allo-SCT, while 34 underwent transplantation with active AML. Not surprisingly, the outcome was extremely poor, with OS and LFS at 3 years of 18% and 9%, respectively. Patients with active disease before transplant had only 3% probability of 3-year OS (71).

Results of allo-SCT were also analyzed by the EBMT on 42 patients, of whom 69% were in first chronic phase, 76% received myeloablative conditioning, and 64% weretransplanted from an MRD. T-cell depletion was applied in 26% and 87% of MRD and MUD, respectively. According to the EBMT risk score (taking into account the patient’s age, disease status, time interval from diagnosis to transplant, donor type, and recipient–donor sex match), 45%, 31%, and 24% of the patients had low, intermediate, and high risk, respectively. This study confirmed the curative potential of allo-SCT in patients with aCML. RFS at 5 years was 36%, NRM was 24%, RR was 40%, and OS was 51%. Age and the EBMT score were significant predictors of OS (72).

Koldehoff et al. reported on 9 patients, of whom 4 were transplanted from MRDs, 4 from MUDs, and 1 from a syngeneic donor. Eight patients received myeloablative conditioning and 8 remain alive, with one relapse of the patient who underwent syngeneic allo-SCT (73). A subsequent follow-up from the same team including 21 patients reported a 5-year OS of 80% with a median survival of 48 months (74). In the early report from MDACC, among 7 patients with aCML, after a median follow-up of 17.5 months, OS and DFS were 35% and 31%, respectively, but five patients died (25). The Japanese group reported outcomes on 19 patients, 15 with aCML and 4 with CNL, who mainly received myeloablative conditioning. One-year OS was >58% and was higher in patients with better performance status and <5% BM blasts (75). Studies reporting results of allo-SCT in patients with aCML are presented in **Table 3**.

**Table 3:** Allogeneic stem cell transplantation in patients with atypical CML.

Author (ref)	No	Donor	Conditioning	Disease phase	PFS	OS
**Lim SN** (41)	**2**	**MRD: 2**	**MAC: 2**	**Chronic: 1 Blastic: 1**	**>100 months**	**>100 months**
**Onida** (72)	**42**	**MRD: 27** **MUD: 15**	**MAC: 32 RIC: 10**	**Chronic: 33 Blastic: 9**	**36% (5-year)**	**51% (5-year)**
**Mittal** (25)	**20**	**MRD: 15** **MUD: 5**	**MAC: 17 RIC: 3**	**NR**	**31% (18-month)**	**35% (18-month)**
**Koldehoff** (73)	**9**	**MRD: 6** **MUD: 3**	**MAC: 8** **RIC: 1**	**NR**	**NR**	**88% (55-month)**
**Koldehoff** (74)	**21**	**NR**	**NR**	**NR**	**NR**	**80% (5-year)**
**Itonaga (** 75)	**14**	**MRD: 5** **MUD: 7** **UCB: 2**	**MAC: 13** **RIC: 1**	**Chronic:9** **Blastic: 5**	**NR**	**54% (1-year)**

MRD, matched related donor; MUD, matched unrelated donor; MAC, myeloablative conditioning; RIC, reduced-intensity conditioning; PFS, progression-free survival; OS, overall survival.

### D. Chronic Neutrophilic Leukemia

CNL is an extremely rare but aggressive disease, with an estimated annual incidence of 1 case per 10,000,000 individuals, has as a diagnostic hallmark various CSF3R mutations and a median life expectancy of about 1.8 years (76). A prognostic model has been developed in the Mayo Clinic based on data from 19 patients. Retrospective analysis from archival material revealed ASXL1 and SETBP1 gene mutations (besides CSF3R) in 47% and 32% of the patients, respectively. Median OS of the whole group was 22.4 months, and CSF3RT618I mutation (present in 14 patients or 74%) was associated with significantly inferior OS compared to truncation mutations (17.2 vs. 42.7 months). On multivariate analysis, ASXL1 mutation, thrombocytopenia (<160 × 10^9^/L), and hyperleukocytosis (>60 × 10^9^/L) were associated with decreased OS, and these 3 parameters created a risk model for prognostic stratification of the patients in high- and low-risk groups (77).

In CNL, AML progression is almost inevitable and occurs at a median of 21 months from initial diagnosis. No standard treatment recommendations exist, and current treatments, mainly consisting of hydroxyurea and interferon-alpha, do not exert any disease-modifying benefit. A recent phase II trial investigating the safety and efficacy of ruxolitinib reported an overall response rate of 35%, making ruxolitinib a promising agent that should be tested in larger patient cohorts (78). Intensive AML-type chemotherapy is usually ineffective when administered after disease progression. Therefore, allo-SCT remains the only potentially curative therapeutic option, and evidence supports early referral as an important factor for better outcome (75, 79). In the largest case series from a nationwide survey in Japan, 5 patients were transplanted between 2003 and 2014. Intention to transplant was based on either disease progression or leukocytosis and splenomegaly uncontrolled by cytoreductive treatment. All patients received myeloablative conditioning, and graft source was an MUD (2), UCB (2), and haploidentical sibling (1). One-year OS was 40%, with one patient dying from sinusoidal obstruction syndrome (d+56), one from bleeding (d+19), and one from disease progression (d+76) (75).

Hydroxyurea or ruxolitinib should be administered to all symptomatic patients or those with significant splenomegaly. Myeloablative conditioning should be administered to fit patients <65 years, while an RIC should be preferred for older or less fit patients. An MRD or MUD should be the preferred option. However, taking into account the recent progress in haplo-SCT in double cord transplants in adults and the experience on the treatment of related disorders, an alternative donor can be used in the absence of a fully matched donor. CSF3R mutation can be used as a marker of an minimal residual disease in the posttransplant period, and persistent detection can alter the adoptive immunotherapy strategy in order to prevent relapse (cyclosporine withdrawal, DLI) (80). Schematically, the treatment algorithm of CNL is shown in **Figure 4**. Studies reporting results on allo-SCT in patients with CNL are presented in **Table 4** (75, 77, 83, 84).

**Table 4:** Allogeneic stem cell transplantation in patients with CNL.

Author (ref)	No	Donor	Conditioning	Disease phase	PFS	OS
**Itonaga** (75)	**5**	**MUD: 2** **UCB: 2** **Haplo: 1**	**MAC: 5**	**Chronic:5**	**NR**	**40% (1-year)**
**Hasle** (81)	**2**	**MRD: 2**	**MAC: 2**	**Chronic:2**	**NR**	**100% (>5-year)**
**Szuber** (77)	**2**	**NR**	**MAC: 2**	**Blastic: 2**	**NR**	**50% (40-month)**
**Goto** (82)	**1**	**MUD: 1**	**MAC: 1**	**Chronic: 1**	**NR**	**100% (3-year)**
**Kako** (83)	**1**	**MUD: 1**	**MAC: 1**	**Blastic: 1**	**Progression d+50**	**NR**
**Piliotis** (84)	**1**	**MRD: 1**	**MAC: 1**	**Chronic:1**	**>1 year**	**>1 year**

MRD, matched related donor; MUD, matched unrelated donor; MAC, myeloablative conditioning; RIC, reduced-intensity conditioning; PFS, progression-free survival; OS, overall survival.

## Factors With Prognostic Importance for Allogeneic Stem Cell Transplantation in Myelodysplastic Syndromes/Myeloproliferative Neoplasms

Many studies, particularly when including several decades of patients, have investigated predictors of outcomes, either simple factors or prognostic tools, evaluating prognosis in the non-transplant setting of the disease. The first EBMT study found that manifestation of grade II–IV acute GVHD and the use of non-T cell-depleted allografts were associated with longer DFS (27). The importance of early transplantation was initially pointed out by the Fred Hutchinson group (31), and when more patients were analyzed, the only variable associated with a higher NRM and shorter OS was the Hematopoietic Cell Transplantation-Specific Comorbidity Index (HCT-CI) (32). The use of posttransplant DLI as an early manipulation of graft failure and of chimerism loss has been applied in at least three studies, two from the Mayo Clinic and one from the King’s College with some successful results reported (26, 30, 33). The latter group has found as important prognostic indicators the percentage of BM blasts (≥5% vs. <5%) and pretransplant cytogenetics (33). Similar results are reported by the more recent analysis from Fred Hutchinson on 85 patients, in which the importance of the HCT-CI was also confirmed. Additional important factors for survival were pretransplant hematocrit and age, whereas the MDAPS (7) and a female donor to female recipient were affecting RR (36).

The group of Milwaukee analyzing 86 transplanted patients with various myeloid malignancies, but none with CMML, found no impact of patient’s obesity on any outcome, although obese patients (Body Mass Index (BMI) >30) had longer hospitalization periods (85). The significance of the chronologic period in which transplantation is performed is easily realizable, since outcomes are improving over time and supportive treatment becomes more effective. Thus, in the French study, major determinants for higher NRM and lower EFS and OS were transplantation before 2004 and the presence of palpable splenomegaly. In the same analysis, female patients exhibited significantly higher RR and NRM, and higher NRM was associated with proliferative CMML and with pretransplant infections (34). The significance of splenomegaly was also stressed by a Chinese study on 25 patients of whom, those with splenomegaly had delayed neutrophil recovery and higher RR and incidence of chronic GVHD (86). In a later report from the Mayo Clinic, splenomegaly, lower HCT-CI, and allo-SCT performed ≤12 months from diagnosis were associated with a more favorable outcome. The small group of MDS/MPN-U, which was analyzed separately, exhibited somewhat better outcomes compared to the outcomes of CMML patients (33). In the EBMT study of 42 aCML patients, patient’s age and the EBMT prognostic score affected OS, whether RFS was higher in MUD, compared to MRD allo-SCT (72).

Increased BM fibrosis has also been recognized as an adverse prognostic factor for DFS and OS, attributed to delayed engraftment, more common cytogenetic abnormalities, and unfavorable driver mutations according to a Chinese retrospective analysis of 239 MDS patients (87). Poor risk cytogenetics and comorbidities were predictors of worse outcome in the retrospective analysis of MDACC on a patient group with MDS and various MDSs/MPNs, exhibiting dismal prognostic factors, for whom RIC was used (88). In the most recent report of the same group focusing on CMML, the outcomes of 83 patients are described, and in multivariate analysis, <5% BM blasts before allo-SCT, the manifestation of chronic GVHD, and initial treatment with HMA were independent predictors of a favorable outcome. In particular, previous treatment with HMA was associated with lower RR and longer PFS (36).

The significance of the donor was investigated on a Japanese group of 159 CMML patients. HLA-matched sibling donor allo-SCT was associated with longer OS and lower NRM, which was highest in the recipients of umbilical cord blood grafts, attributed to delayed neutrophil engraftment (39). In the study from Heidelberg, unrelated donor allo-SCT and TBI-included conditioning were associated with better OS, whereas CPSS could nicely stratify the probability of OS. In this analysis, age was not a significant factor for OS and no clear benefit was proven for transplanted patients with lower-risk CPSS over those not transplanted. However, as in other studies, CPSS could not predict NRM (42). The impact of GVHD was investigated by two Japanese studies of 115 and 141 patients, respectively. In the first, CMML and Refractory anemia with Excess of Blasts (RAEB) patients were pooled together (44 with CMML). An RIC regimen was given to 70% of the patients, and although many of them were older with poor cytogenetics, exhibited similar 4-year OS. Factors associated with poorer survival were poor cytogenetics, BM blasts ≥20% at transplantation, and absence of chronic GVHD, whereas for high-risk patients, the manifestation of chronic GVHD was associated with longer survival (89). In the second study, analysis has been focused on successfully engrafted CMML patients. Grade I acute GVHD was related to better OS and lower leukemia-related death in univariate analysis, whereas in multivariate analysis, extensive chronic GVHD was associated with significantly better OS and lower leukemia-related death in patients who were not transplanted in CR (90). In the IBMTR study, CPSS could only predict OS, which was better in the Low/Intermediate-1 group, and patients with higher CPSS had about twice as high risk for post-relapse death. On multivariate analysis, performance status, CPSS, and the type of graft were independent predictors of DFS and OS (47). In the EBMT study, besides the impact of the period of transplantation, factors associated with a longer PFS and OS were disease status at transplantation (CR vs. no CR) and shorter interval from diagnosis to allo-SCT. Patients transplanted in CR had lower probability for NRM, and disease status at transplantation was the only significant factor for RFS and OS in multivariate analysis (48).

The significance of specific mutations was initially investigated by a German group on 45 patients who were screened for ASXL1, CBL, NRAS, and TET2 gene mutations. ASXL1 and TET2 were the most commonly mutated genes, but the type and the number of mutations had no impact on any outcome. The presence of mutations was used as a marker of minimal residual disease, and their persistence at 6 months posttransplant was associated with higher RR (37). Similar results were obtained by a Chinese study on 59 CMML patients in which the significance of post-allo-SCT minimal residual disease detected by both, flow cytometry and by PCR of the WT1 gene was investigated. Both techniques demonstrated a high level of prognostic value and could predict posttransplant relapse. The significance of the presence of WT1 mutations was not investigated (91).

The Mayo Clinic group compared 4 different prognostic scores and various clinical and genetic factors, including common mutations, on 70 transplanted and in 336 non-transplanted patients. Allo-SCT in other than chronic phase, abnormal cytogenetics, and neutropenia <1.5 × 10^9^/l were predictors of worse outcome. No prognostic score or any mutation had any impact on transplantation outcome. Patients with proliferative type had significantly longer survival when transplanted compared to those who were not transplanted (50 vs. 19 months), whereas a similar difference was not observed among patients with the dysplastic type (23, 24).

In contrast, the Nordic group found that TET2 mutations were associated with a favorable outcome, whereas ASXL1, RUNX1, and RAS mutations were associated with worse OS. Transfusion dependency and higher WBC count before transplant were also associated with earlier relapse, and NRAS mutations were linked to poorer survival due to increased TRM (43). In the more recent analysis of Fred Hutchinson, relapse was associated with poor cytogenetics, higher CPSS and MDACC score, and the presence of pretransplant residual disease, whereas death was associated with poor cytogenetics, pretransplant residual disease, and high HCT-CI. Clonal mutations were identified in 40.3% of the patients, and WT1 and ATRX mutations were associated with a higher RR and a shorter OS. NRAS and a high number of mutations (>10 in general or >4 epigenetic mutations) were also associated with higher RR (40).

Finally, in a recently published cooperative study on 240 CMML patients with a long median follow-up, increased percentage of BM blasts (>2%), the HCT-CI, and mutations of the ASXL1 or the KRAS genes were found to retain independent prognostic significance for OS and RFS. Collecting these factors, the authors have introduced the first prognostic tool that addresses specifically CMML patients to be transplanted (CMML-specific transplantation-specific prognostic score). The score ranges between 0 and 20 and assigns 1 point to each of the 8 comorbid conditions, described by the HCT-CI, and 4 points to each of the following three factors: pretransplant BM blasts >2 and the presence of ASXL1 or NRAS mutations. This score was superior to any previously reported, nicely predicted NRM and OS by stratifying the patients in 5 groups, with 5-year OS ranging between 19% and 81%, but has not yet been prospectively validated (50). **Table 5** summarizes the factors that have been shown to impact prognosis in CMML patients either following a non-transplant approach or undergoing allo-SCT.

**Table 5:** Well-recognized prognostic factors.

At initial diagnosis	At transplantation
**Age**	**Age**
**HCT Comorbidity Index**	**HCT Comorbidity Index**
**Performance status**	**Performance status at transplantation**
**RBC transfusion dependency**	**RBC transfusion dependency**
**Palpable splenomegaly**	**Palpable splenomegaly**
**Lymphadenopathy and other extramedullary disease**	**Lymphadenopathy and other extramedullary disease**
**Cytogenetic abnormalities IPSS-described**	**Cytogenetic abnormalities IPSS-described**
**Presence of constitutional symptoms**	**Disease status at transplantation**
**Gene mutations (ASXL1, RUNX1, SRSF2, JAK2, NRAS vs. TET2)**	**Gene mutations (ASXL1, RUNX1, SRSF2, JAK2, NRAS vs. TET2)**
**Trisomy 8**	**Number of genes mutated**
**FAB subtype (Dysplastic or Proliferative)**	**FAB subtype (Dysplastic or Proliferative)**
**Leukocytosis >15 x 10^9/^l/Unstable leukocytosis**	**Leukocytosis > 15 × 10^9/^l**
**Severity of monocytosis**	**Severity of monocytosis**
**Peripheral blood lymphocytosis**	**Time interval from diagnosis to transplantation**
**Presence of circulating blast cells**	**Use of HMA as bridging treatment**
**Anemia <10 g/dl or severe anemia <8 g/dl**	**Presence of circulating blast cells**
**Thrombocytopenia <100 × 10^9^/l**	**Anemia <10 g/dl or severe anemia <8 g/dl**
**Bone Marrow blasts ≥5%**	**Neutropenia <1.5 × 10^9^/l**
**Elevated serum LDH**	**Thrombocytopenia <100 × 10^9^/l**
**Elevated serum ferritin levels**	**Bone Marrow blasts ≥5%**
	**Active pretransplant infections**
**Specific prognostic tools (CPSS, CPSS-mol, Mayo Prognostic Model, MDACC Index, etc.)**	**CPSS**
	**MD Anderson Prognostic Index**
	**EBMT Prognostic Score**
	**Increased Bone marrow fibrosis**
	**Myeloablative conditioning**
	**Use of non-T cell-depleted grafts**
	**Donor HLA matching**
	**Donor sex mismatch**
	**Development of acute GVHD (unfavorable)**
	**Development of chronic GVHD (favorable)**

## Discussion—Conclusions and Current Recommendations

CMML and other overlap MDSs/MPNs are challenging therapeutic problems for the treating physician. As a result of the substantial disease heterogeneity, he or she has to correctly identify the profile of the risk factors in each individual patient, evaluate his or her health background, and appropriately design the interventional treatment approach (15, 16, 92). For patients younger than 60 and for those older than 70 years, such approaches are rather easy to be designed, since by now the only curative intervention remains allo-SCT, which cannot be applied to very elderly and frail patients (16, 18, 92, 93). The most challenging decision for the treating physician concerns patients between 60 and 70 years and few fit patients older than 70 years. For this age range, the physician needs to discriminate higher-risk features that have been well characterized and described (10, 11). A disease mutational profile can greatly help in any case but particularly for patients of the seventh decade of their life (20). Ideally, all patients with CMML and adverse prognostic features and all patients with other MDSs/MPNs, securing an available stem cell donor should proceed to allo-SCT. Among the adverse prognostic features, WHO classification-defined CMML-1 or CMML-2, proliferative type of disease with difficult control of leukocytosis, presence of splenomegaly, extramedullary disease, constitutional symptoms, elevated serum LDH and an otherwise unexplained proinflammatory profile, manifestation of transfusion dependency, thrombocytopenia and increased marrow fibrosis, adverse cytogenetics and/or mutation profile, and a high CPSS or other relevant prognostic score are included (8–11). When eligible patients have been identified, they should thoroughly be informed as early as possible and consent for the recommended approach should be obtained.

When the basic plan has been organized, there are some “technical issues” that should be resolved. Regarding the best timing, patients exhibiting the previously mentioned profile should rapidly undergo allo-SCT after a few months of bridging treatment. The kind of this treatment (AML-type chemotherapy or HMA) appears not to play a core role, although there is emerging evidence that HMA should be preferred in CMML patients of more advanced age and cytoreductive treatment should be the option for younger CMML patients and for those with other MDSs/MPNs (36, 92, 93). For patients with an excess (>5%) of marrow blasts, achieving a CR before transplant with the bridging treatment appears to favor a better posttransplant outcome (18, 22, 40, 43, 49). Another important “technical” issue is identification of the appropriate donor. Although data analysis from several studies has not found any significant impact of the type of donor on the outcome, it appears that this may be valid for relatively younger patients. For patients with more advanced age, the identification of a fully matched donor secures a clearly better outcome (40). A third “technical” issue concerns the use of the appropriate conditioning regimen.

**Figure 1:**
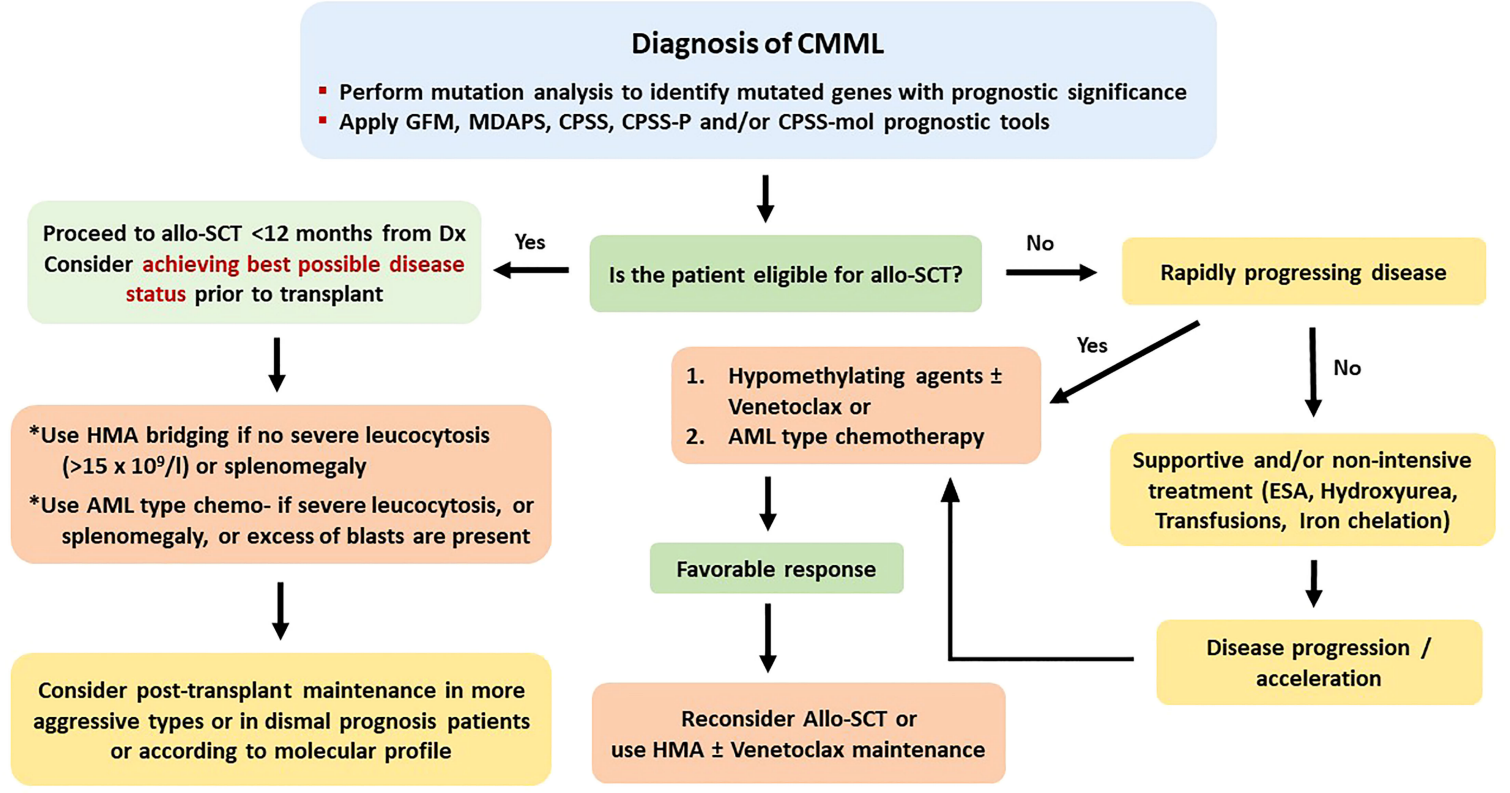
Recommended treatment algorithm for patients with chronic myelomonocytic leukemia.

**Figure 2:**
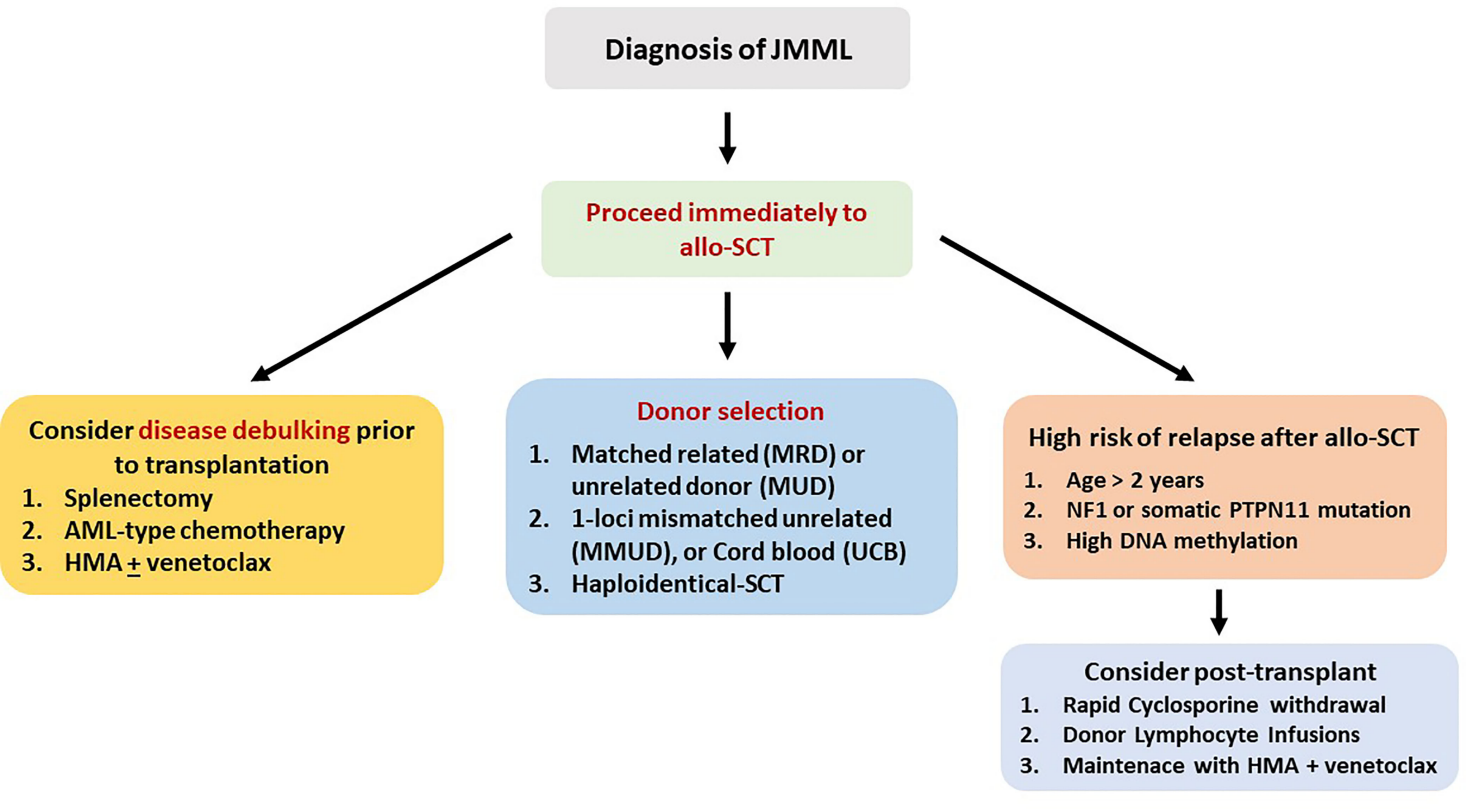
Recommended treatment algorithm for patients with juvenile myelomonocytic leukemia. All patients should be considered candidates for allo-SCT.

**Figure 3:**
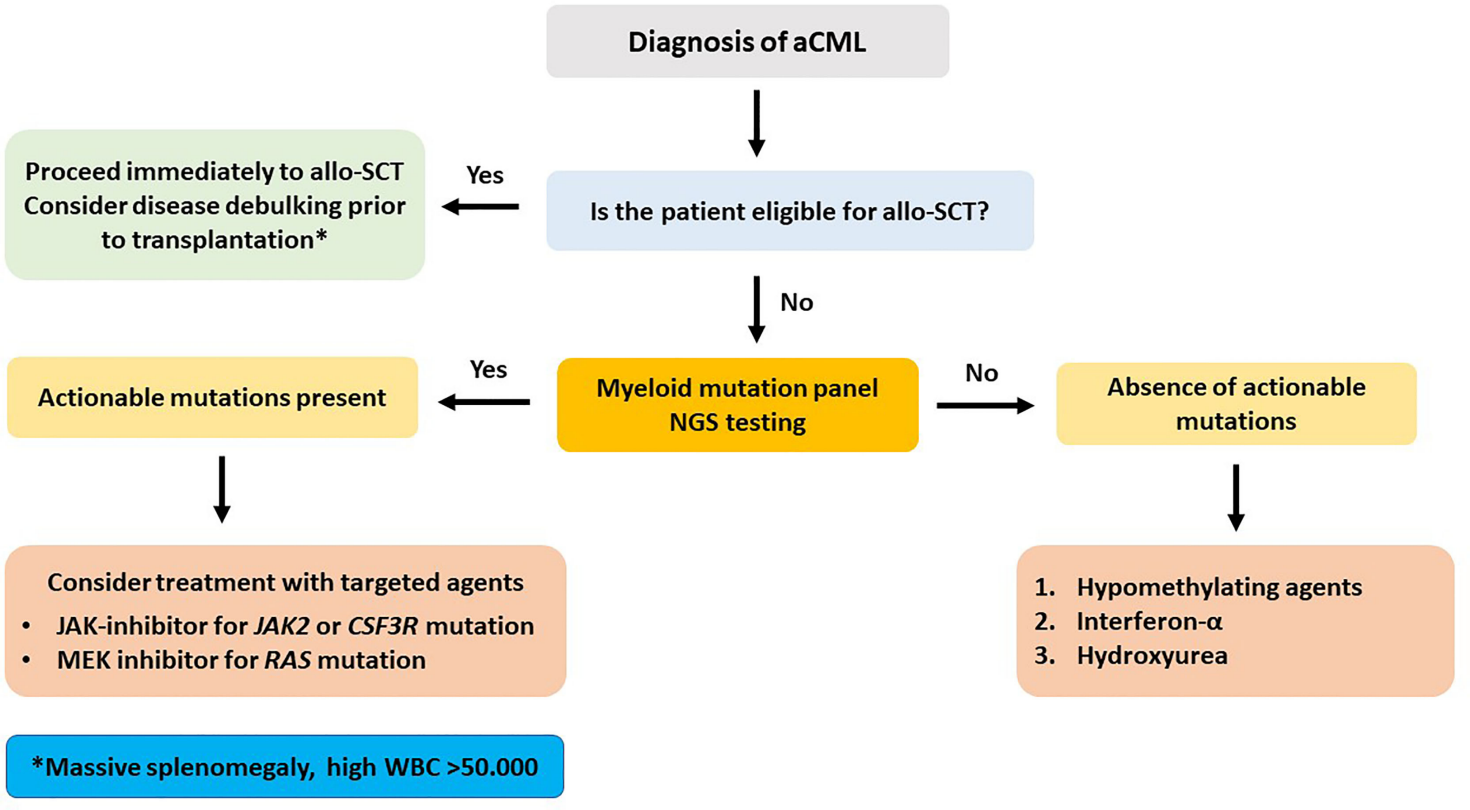
Recommended treatment algorithm for patients with atypical chronic myelogenous leukemia. Massive splenomegaly and extreme leukocytosis at the time of initial diagnosis bear a severely dismal prognosis.

**Figure 4:**
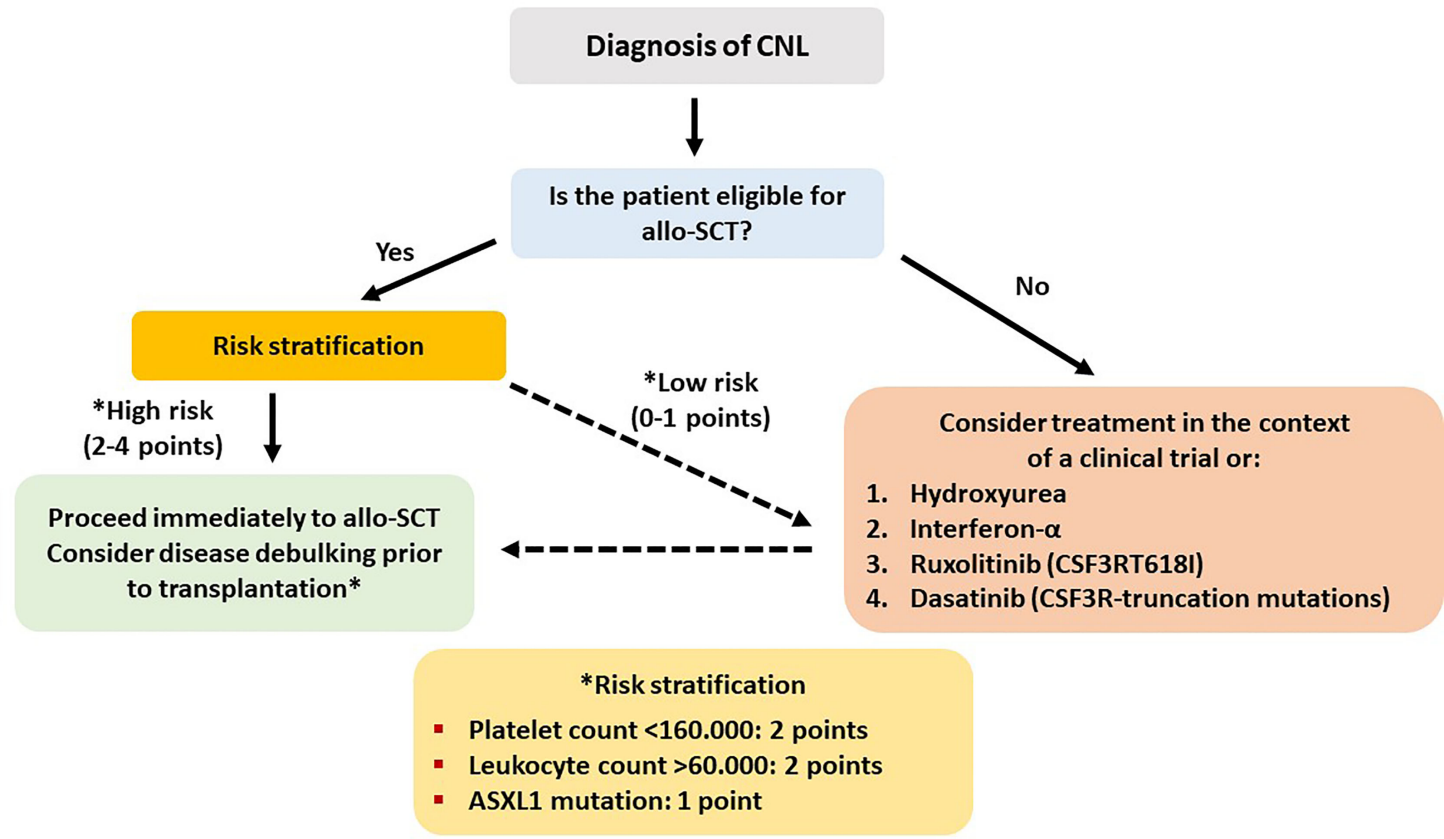
Recommended treatment algorithm for patients with chronic neutrophilic leukemia.

For the abovementioned issues, the following basic principles can be applied. 1) Myeloablative conditioning should be preferred in younger and fit patients, while for older patients above the age of 65 years or for those with significant comorbidities, an RIC regimen appears to be more suitable. 2) A matched related or unrelated donor should be used, but in the absence of an available matched donor, haploidentical or cord blood transplantation should be considered at least for patients younger than 60 years. 3) Cytoreductive or HMA treatment should be administered in symptomatic patients and in those with splenomegaly or with CMML-1/2 before allo-SCT. For patients without an excess of marrow blasts, HMA bridging treatment might suffice. Evaluating patient risk category contributes to better predict several outcomes. To this endpoint, some of the described adverse prognostic factors might indeed reflect other already known prognostic factors. For example, in CMML, splenomegaly and leukocytosis apparently reflect a proliferative type of the disease, whereas circulating blasts and BM blasts >5% apparently fit with WHO-defined CMML-1 or CMML-2. The prognostic ability of CPSS in CMML is a debate. Some authors found it to be predictable, whereas other did not (23, 24, 35, 47). However, newer prognostic tools have been proposed, such as the CPSS-mol, the CPSS-P, and the CPSS transplant-specific, which have incorporated the prognostic importance of mutations that can predict outcome in transplanted patients, as this has been shown in several retrospective studies (23, 50, 74, 91, 93). Using these tools might help to better distinguish the transplantation risk group. The potentially ideal diagnostic and therapeutic recommendations for the four different types of myeloid neoplasia, for which this review is dedicated (CMML, aCML, JMML, and CNL), according to the authors’ opinion are shown in **Figures 1**–**4**, respectively.

Probably the most important parameter for a successful transplantation is to help the patients achieve the best possible disease status before transplantation. To this point, there are newer targeted treatments besides HMA, which have not yet been tested as treatment tools. These include ruxolitinib and other JAK inhibitors, the CPX-351 complex, RAS and Hedgehog pathway inhibitors, the newer approved oral combination decitabine/cedazuridine, venetoclax, IDH1/IDH2 inhibitors, and other agents. The application of these agents might induce a better remission status before transplantation, thus rendering allo-SCT more effective. However, establishing the most appropriate drug combinations in each individual patient is a long way, which could be delineated through the use of these combinations either as a bridging treatment before transplantation or by incorporating appropriate drugs in the preparatory conditioning regimens. All of these potential new directions could only be substantiated through prospective multicenter randomized trials.

## Author Contributions

AS guided the article. AS and PT designed the article, wrote the main parts of the article, and critically reviewed the relevant literature. SC, VL, EV, and AK performed literature search, wrote parts of the article, and contributed to the design of the Tables and Figures. All of the authors reviewed and approved the final version of the article.

## Funding

The University of Patras, through a personal account of the first author has covered the publication fees of this article.

## Conflict of Interest

The authors declare that the research was conducted in the absence of any commercial or financial relationships that could be construed as a potential conflict of interest.

## Publisher’s Note

All claims expressed in this article are solely those of the authors and do not necessarily represent those of their affiliated organizations, or those of the publisher, the editors and the reviewers. Any product that may be evaluated in this article, or claim that may be made by its manufacturer, is not guaranteed or endorsed by the publisher.

## Abbreviations

aCML, atypical bcr/abl-negative chronic myeloid leukemia; allo-SCT, allogeneic hematopoietic stem cell transplantation; AML, acute myelogenous leukemia; ATG, anti-thymocyte globulin; BM, bone marrow; CMML, chronic myelomonocytic leukemia; CNL, chronic neutrophilic leukemia; DFS, disease-free survival; DLI, donor lymphocyte infusion; EBMT, European Blood and Marrow Transplantation Registry; GVHD, graft-versus-host disease; GVL, graft-versus-leukemia; HbF, fetal hemoglobin; HCT-CI, Hematopoietic Cell Transplantation-Specific Comorbidity Index; HMA, hypomethylating agent; HLA, human leukocyte antigen; JMML, juvenile myelomonocytic leukemia; IBMTR, International Blood and Marrow Transplantation Registry; LDH, lactate dehydrogenase; LFS, leukemia-free survival; MDACC, MD Anderson Cancer Center; MDAPS, MD Anderson Prognostic Score; MDS/MPN, mixed or hybrid or overlap myelodysplastic syndrome/myeloproliferative neoplasm; MDS/MPN-U, myelodysplastic syndrome/myeloproliferative neoplasm unclassifiable; MMUD, mismatched unrelated donor; MPN, myeloproliferative neoplasm; MRD, matched related donor; MUD, matched unrelated donor; NRM, non-relapse mortality; OS, overall survival; PB, peripheral blood; PFS, progression-free survival; RAB, refractory anemia with an excess of blasts; RR, relapse rate; TBI, total body irradiation; TLI, total lymphoid irradiation; TRM, treatment-related mortality; UCB, umbilical cord blood; WBC, white blood cell; WHO, World Health Organization.

## Glossary

**Table:**

aCML	atypical bcr/abl-negative chronic myeloid leukemia
aGVHD	acute Graft Versus Host Disease
allo-SCT	allogeneic hematopoietic stem cell transplantation
AML	acute myelogenous leukemia
ASXL1	Additional Sex Combs Like-1 gene
ATG	anti-thymocyte globulin
ATRX	Alpha Thalassemia mental Retardation X-linked helicase
BCL2	B-Cell Lymphoma type 2 gene
BM	bone marrow
BMI	Body Mass Index
BP	Blastic Phase
BRAF	B-Raf protooncogene, serine/threonine kinase
CALR	Calreticulin
CBL	Cbl protooncogene, E3ubiquitin kinase
cGVHD	chronic Graft Versus Host Disease
CSFR3	ColonyStimulating Factor-3 Receptor
CMML	chronic myelomonocytic leukemia
CNL	chronic neutrophilic leukemia
CPSS	CMML-specific Prognostic Scoring System
CPSS-mol	CPSSmolecular
CPSS-P	CPSS including platelet count
CR	Complete Remission
DFS	disease-free survival
DLI	donor lymphocyte infusion
DNA	DeoxyRibonucleotidic Acid
EB	Excess of Blasts
EBMT	European Blood and Marrow Transplantation Registry
EFS	Event-Free Survival
ETNK1	Ethanolamine kinase 1
EWOG-MDS	European Working Group on MDS
EZH2	Enhancer of Zeste 2 Polycomb Repressive Complex 2
GVHD	graft-versus-host disease
GVL	graft-versus-leukemia
HbF	fetal hemoglobin
HCT-CI	Hematopoietic Cell Transplantation-Specific Comorbidity Index
HMA	hypomethylating agent
HLA	human leukocyte antigen
JAK2	Janus Kinase type 2
JMML	juvenile myelomonocytic leukemia
IBMTR	International Blood and Marrow Transplantation Registry
K-M	Kaplan-Meier
KRAS	KRAS protooncogene GTPase
LDH	lactate dehydrogenase
LFS	leukemia-free survival
MDACC	MD Anderson Cancer Center
MDAPS	MD Anderson Prognostic Score
MDS/MPN	mixed or hybrid or overlap myelodysplastic syndrome/myeloproliferative neoplasm
MDS/MPN-U	myelodysplastic syndrome/myeloproliferative neoplasm unclassifiable
MEK	Mitogen-activated protein Kinase kinase
MMUD	mismatched unrelated donor
MPL	MPL protooncogene, thrombopoietin receptor
MPN	myeloproliferative neoplasm
MRD	matched related donor
MUD	matched unrelated donor
NF1	Neurofibromatosis type 1 gene
NRAS	NRAS protooncogene GTPase
NRM	non-relapse mortality
OS	overall survival
PB	peripheral blood
PCR	Polymerase Chain Reaction
PFS	progression-free survival
PTPN11	Protein Tyrosin Phosphatase Non-receptor 11
RAEB	refractory anemia with an excess of blasts
RAF	Rapidly Accelerated Fibrosarcoma
RAS	Rat Sarcoma gene
RFS	Relapse-free survival
RIC	Reduced Intensity Conditioning
RR	relapse rate
RUNX1	Runt-related transcription factor 1
SCT	Stem cell transplantation
SETBP1	SET binding protein 1
SOS1	SOS Ras/Rac guanine nucleotide exchange factor 1
SRSF2	Serine andarginine Rich Splicing Factor 2
TBI	total body irradiation
TET2	Ten-Eleven Translocation type 2 gene
TLI	total lymphoid irradiation
TRM	treatment-related mortality
UCB	umbilical cord blood
USA	United States of America
WBC	white blood cell
WHO	World Health Organization
WT1	Wilms Tumor 1 gene
